# Pregabalin Monotherapy for Chronic Pain Associated with Osteoarthritis in Working Dogs: A Retrospective Study of 21 Cases

**DOI:** 10.3390/ani16203186

**Published:** 2026-10-11

**Authors:** Jungmo Lee, Gyeonggook Park, Junseok Yoo, Do-gyoon Ahn, Inhyung Lee

**Affiliations:** 1 Department of Veterinary Clinical Sciences, College of Veterinary Medicine, Seoul National University, Seoul 08826, Republic of Korea; jmlee6767@naver.com; 2 Department of Veterinary Medicine, Armed Forces Medical Research Institute, Daejeon 34059, Republic of Korea; gghk0924@gmail.com (G.P.); js3685@naver.com (J.Y.); gta3721@naver.com (D.-g.A.)

**Keywords:** working dogs, osteoarthritis, chronic pain, pregabalin, Liverpool Osteoarthritis in Dogs, LOAD, Canine Brief Pain Inventory, CBPI, clinical metrology instrument

## Abstract

Degenerative joint disease, including osteoarthritis (OA), is frequently diagnosed in working dogs and is a major cause of retirement or euthanasia. Pregabalin is a gabapentinoid used mainly for neuropathic pain, but evidence for pregabalin monotherapy in canine OA-related chronic pain is limited. Of 702 cases screened, this retrospective study included 21 working dogs with radiographically confirmed OA and chronic pain of at least three months’ duration that received pregabalin monotherapy (4 mg/kg PO q12h). Handler-completed Liverpool Osteoarthritis in Dogs (LOAD), Canine Brief Pain Inventory (CBPI) pain severity score (PSS), and pain interference score (PIS) at baseline and at 14 ± 3 days were compared using paired *t*-tests, with predefined responder criteria applied to individual dogs. LOAD, CBPI-PSS, and CBPI-PIS decreased by 2.33, 0.67, and 1.00 points, respectively (all *p* < 0.001). However, only 5 of 21 dogs (23.8%) met the LOAD responder criterion and 3 of 21 (14.3%) met the CBPI criterion. No adverse effects were recorded. Although scores decreased significantly at the group level, clinically meaningful improvement was limited, and the uncontrolled design precludes causal inference. Controlled studies are needed to determine whether pregabalin has a role when non-steroidal anti-inflammatory drug use requires caution.

## Simple Summary

Working dogs, including military and police dogs, can develop osteoarthritis, a painful degenerative joint disease that may reduce mobility, daily activity, and working ability. Anti-inflammatory pain medicines are commonly used, but stomach and kidney problems may limit their use in some dogs. Medical records of 21 working dogs with osteoarthritis-related chronic pain that received pregabalin as their only pain medicine were reviewed. Pregabalin changes pain signaling in the nervous system rather than directly reducing inflammation. Handlers rated each dog’s pain and daily function before treatment and again about two weeks later using two established questionnaires. On average, pain and function scores were lower at the second assessment than before treatment, and no adverse effects were recorded in the medical records. However, only a small number of dogs improved enough to reach a level considered meaningful in daily life. Because the study had no comparison group, these changes cannot be attributed to pregabalin itself. Whether pregabalin is useful for dogs in which anti-inflammatory pain medicines must be used with caution should be tested in larger controlled studies.

## 1. Introduction

Degenerative joint disease, including osteoarthritis (OA), is one of the most frequently diagnosed disease groups in working dogs [1]. It has also been reported as one of the major reasons for retirement and euthanasia in military and other working dogs [2,3,4].

OA-associated pain is multifactorial and is not necessarily proportional to the degree of structural joint degeneration [5]. Peripheral nociceptive input driven by mechanical loading and inflammatory mediators is considered the main source of OA pain, and peripheral and central sensitization may further amplify and maintain it [6,7,8]. These pain mechanisms can reduce the activity level and quality of life of affected dogs [6] and may impair the operational performance of working dogs. Therefore, long-term pain management and the preservation of function are important therapeutic goals in dogs with OA.

Pregabalin is a gabapentinoid that is used in dogs mainly for the control of neuropathic pain [9]. Like gabapentin, pregabalin binds to the α_2_δ-1 subunit of voltage-gated calcium channels [9]. Following nerve injury, pore-forming calcium channel subunits, mainly of the N-type, are trafficked to presynaptic terminals in the spinal dorsal horn; gabapentinoids inhibit this anterograde trafficking and thereby reduce calcium influx and the release of excitatory neurotransmitters [10,11]. Additional mechanisms have been proposed, including interactions with N-methyl-D-aspartate receptors and transient receptor potential channels, modulation of supraspinal noradrenergic pathways, and effects on cytokines and protein kinase C [10,11,12]. Because these actions target neuronal excitability and sensitization rather than joint inflammation, gabapentinoids are considered most relevant when sensitization or a neuropathic component contributes to chronic pain [13].

In dogs, pregabalin is rapidly absorbed after oral administration, and a median elimination half-life of 6.9 h (range 6.21–7.40 h) has been reported after a single oral dose of approximately 4 mg/kg [14]. On the basis of these single-dose data, it was predicted that 4 mg/kg administered every 12 h would maintain plasma concentrations within the therapeutic range proposed in humans [14]. However, these predictions were derived from a single-dose study in a small number of healthy dogs, and the therapeutic range was extrapolated from human data. Importantly, pharmacodynamic studies of pregabalin are lacking in dogs, and the plasma concentrations required for analgesia have not been established. Therefore, these pharmacokinetic data support the feasibility of repeated twice-daily administration but do not provide evidence of clinical efficacy in OA. Clinical evidence for gabapentinoids in canine OA-related chronic pain is limited, and studies evaluating pregabalin monotherapy in particular are scarce [6,15].

The aim of this retrospective study was to evaluate changes in pain- and function-related clinical metrology scores before and after initiation of pregabalin monotherapy in working dogs with OA-associated chronic pain. For this purpose, two validated owner-completed clinical metrology instruments (CMIs), the Liverpool Osteoarthritis in Dogs (LOAD) instrument and the Canine Brief Pain Inventory (CBPI), were used [16,17]. Specifically, it was assessed (1) group-level changes from baseline to follow-up in LOAD and in the CBPI pain severity score (PSS) and pain interference score (PIS), and (2) the proportion of individual dogs that met predefined criteria for a clinically meaningful response. It was hypothesized that LOAD, CBPI-PSS, and CBPI-PIS scores would decrease from baseline to follow-up.

## 2. Materials and Methods

### 2.1. Animals and Study Design

This was a retrospective chart review study based on the electronic medical records of working dogs that presented to the Armed Forces Medical Research Institute, were diagnosed with OA, and were prescribed pregabalin (4 mg/kg PO q12h) for the management of chronic pain. All cases recorded between June 2025 and June 2026 were screened, and all cases that met the eligibility criteria were included; no a priori sample size calculation was performed.

No intervention, additional diagnostic testing, or other experimental procedures were performed for the purpose of this study, and only available clinical data were used.

### 2.2. Inclusion and Exclusion Criteria

Eligible cases in this study were working dogs that presented with chronic pain or gait abnormality as the primary complaint and were subsequently diagnosed with OA radiographically. OA-associated chronic pain was diagnosed during routine clinical care on the basis of a combination of clinical and radiographic findings: (1) a history of pain or gait abnormality obtained from the handler; (2) pain on palpation or manipulation of the affected joints, reduced range of motion, and lameness identified on physical examination by veterinarians at the Armed Forces Medical Research Institute; and (3) radiographic evidence of OA in the appendicular joints. In accordance with the definition of chronic pain, only cases in which pain or gait abnormality had persisted for at least three months were included [18].

To minimize confounding factors that could influence pain assessment and to evaluate changes in clinical measures related to OA, dogs with concurrent conditions other than OA that could affect the pain response or gait were excluded. These conditions included neurological diseases, such as intervertebral disc disease and degenerative lumbosacral stenosis; other musculoskeletal diseases, such as fractures and muscle or ligament injuries; prostatic diseases; and neoplastic diseases [19,20,21]. These conditions were identified from the diagnoses documented in the medical records.

Cases that received concurrent analgesic, anti-inflammatory, or adjunctive analgesic agents other than pregabalin, or that had a record of such medication within two weeks before the baseline assessment, were excluded. These agents included non-steroidal anti-inflammatory drugs (NSAIDs), corticosteroids, opioids (including tramadol), other gabapentinoids, and amantadine. The two-week interval was intended to minimize carry-over effects of previous treatment on the baseline assessment. Dogs in which the prescribed pregabalin dose differed from 4 mg/kg PO q12h for clinical reasons, such as behavioral disorders or neurological diseases, were also excluded. None of the included dogs received physiotherapy, rehabilitation, acupuncture, nutraceutical supplements, or a structured weight-management program during the observation period.

Cases were included in the analysis only if both LOAD and CBPI scores, with the PSS and PIS recorded separately, were documented at baseline and at a follow-up assessment within the predefined window (Section 2.4); missing data were not imputed.

### 2.3. Radiographic Evaluation and OA Grading

Diagnosis of OA and assessment of radiographic severity were performed with radiography of the appendicular joints. Radiographic OA severity was assessed by applying a modified Kellgren–Lawrence grading system that has been used for canine stifle OA [22,23,24] and was routinely applied at this institution during the study period. The presence and extent of osteophyte formation, subchondral sclerosis, roughness of the bone surface, and bony deformity were considered during assessment.

OA lesions in each joint were classified from grade 0 to grade 4 according to the degree of radiographic change. Grade 0 was defined as the absence of radiographic OA change, and increasing grades reflected increases in the size and number of osteophytes and in the severity of subchondral sclerosis and bony deformity [22,23,24]. As part of routine practice, radiographs were reviewed by two veterinarians, each of whom determined the presence or absence of OA and assigned a radiographic grade to each joint. These findings were discussed at the case rounds held on the day after the examination; in cases of disagreement, either in the determination of OA presence or in the assigned grade, the radiographs were jointly re-evaluated until a consensus was reached, and only the final consensus grade was recorded in the medical records. When OA lesions were identified in two or more joints of the same dog, the grade of the joint showing the highest radiographic grade was defined as the final OA grade.

### 2.4. Clinical Pain Assessment and Data Collection

Breed, sex, age, pattern of joint involvement (monoarticular or polyarticular), final OA grade, pregabalin dose and duration of administration, LOAD and CBPI assessment records, and treatment changes made at the follow-up visit were collected from the medical records of the selected dogs.

Clinical pain and function were assessed using LOAD and the CBPI, which are CMIs developed and validated for use by owners; in this study, the questionnaires completed by the handler of each dog were analyzed [16,17]. LOAD was used to assess OA-related functional impairment, such as activity level, exercise ability, lameness, and stiffness, with a total score ranging from 0 to 52, in which higher scores indicate more severe functional impairment [16]. The CBPI was used to assess the severity of pain and its impact on daily function through the CBPI-PSS and CBPI-PIS, respectively; the PSS was calculated as the mean of four items and the PIS as the mean of six items, each rated on a 0–10 scale [17]. Both questionnaires were routinely completed as part of clinical care for dogs presenting with OA. Baseline questionnaires were completed at the initial visit, before the first dose of pregabalin, and follow-up questionnaires were completed at the recheck visit. Handlers were not blinded to treatment.

Baseline and follow-up assessments for a given dog were included only when both had been completed by the same handler. This criterion was applied to minimize inter-rater variability and improve the consistency of within-dog comparisons over time. Baseline LOAD, CBPI-PSS, and CBPI-PIS scores and the corresponding follow-up scores were collected. The baseline and follow-up clinical examinations were not necessarily performed by the same veterinarian; however, because the outcomes analyzed in this study were the handler-completed LOAD and CBPI scores, the examining veterinarian did not contribute to the outcome measures.

The target follow-up time point was set at 14 days after the start of pregabalin administration. This was based on the design of a previous clinical study of canine OA in which the response to analgesic treatment was evaluated using changes in PSS and PIS between baseline and day 14 [17,25]. In addition, because LOAD contains items relating to activity level, exercise ability, lameness, stiffness, and the influence of weather [16], it was considered that over longer assessment intervals, changes in activity level, training intensity, weather, and other environmental factors could influence the assessment results. Conversely, a very short interval might not allow treatment-related changes in chronic pain and function to become consistently apparent and might increase the influence of day-to-day fluctuations. Accordingly, the target follow-up time point was set at 14 days to reduce heterogeneity in assessment timing between cases and to ensure the consistency of the data. Considering the retrospective nature of the study, follow-up assessments performed between days 11 and 17 after the start of treatment (14 ± 3 days) were accepted.

Adverse effects were identified retrospectively by reviewing all medical records from the initiation of pregabalin to the follow-up assessment. Clinical signs including sedation, ataxia, decreased appetite, vomiting, and diarrhea were sought in the free-text entries of the clinical records; no predefined questionnaire or checklist was used.

### 2.5. Statistical Analysis

All statistical analyses were performed using Python (version 3.13.5; Python Software Foundation, Wilmington, DE, USA).

Continuous variables were summarized as mean ± standard deviation (SD) or as median and interquartile range (IQR), and categorical variables were presented as numbers and percentages. The change in CMI score was calculated as the follow-up value minus the baseline value, so that a negative change indicated improvement in the clinical score.

LOAD, CBPI-PSS, and CBPI-PIS were defined as co-primary outcomes because they capture complementary aspects of OA-related chronic pain, namely function, pain severity, and pain interference [16,17]. For each outcome, normality of the within-subject paired differences was assessed using the Shapiro–Wilk test. Baseline and follow-up scores were compared using two-sided paired *t*-tests, and mean changes were presented with 95% confidence intervals (CI). Because of the small sample size, the Wilcoxon signed-rank test was additionally performed as a non-parametric sensitivity analysis. To account for multiplicity across the three co-primary outcomes, a Bonferroni-adjusted significance level of *p* < 0.0167 (0.05/3) was applied. The effect size of within-subject change was calculated as Cohen’s *d_z_* for paired samples, obtained by dividing the mean paired change by the standard deviation of the paired differences.

To evaluate clinically meaningful change in addition to statistical significance, predefined responder criteria were applied to individual dogs. For LOAD, the responder criterion was defined as a decrease of at least 4 points from baseline, corresponding to the published minimal clinically important difference [26,27]; for the CBPI, the responder criterion was defined as a decrease of at least 1 point in PSS together with a decrease of at least 2 points in PIS from baseline [25]. Dogs meeting each criterion were classified as responders, and the frequency of responders was presented as numbers and percentages.

In the exploratory subgroup analyses, between-group comparisons of CMI changes were performed using the Mann–Whitney U test. Associations between age or OA grade and changes in LOAD, CBPI-PSS, and CBPI-PIS were assessed using Spearman’s rank correlation.

All statistical tests were two-sided. Except for the Bonferroni-adjusted co-primary comparisons, *p* < 0.05 was considered statistically significant. Subgroup and correlation analyses were performed as exploratory analyses, and no correction for multiple comparisons was applied to them; their results should therefore be interpreted as hypothesis-generating.

### 2.6. Use of Generative Artificial Intelligence

**Figure 1:**
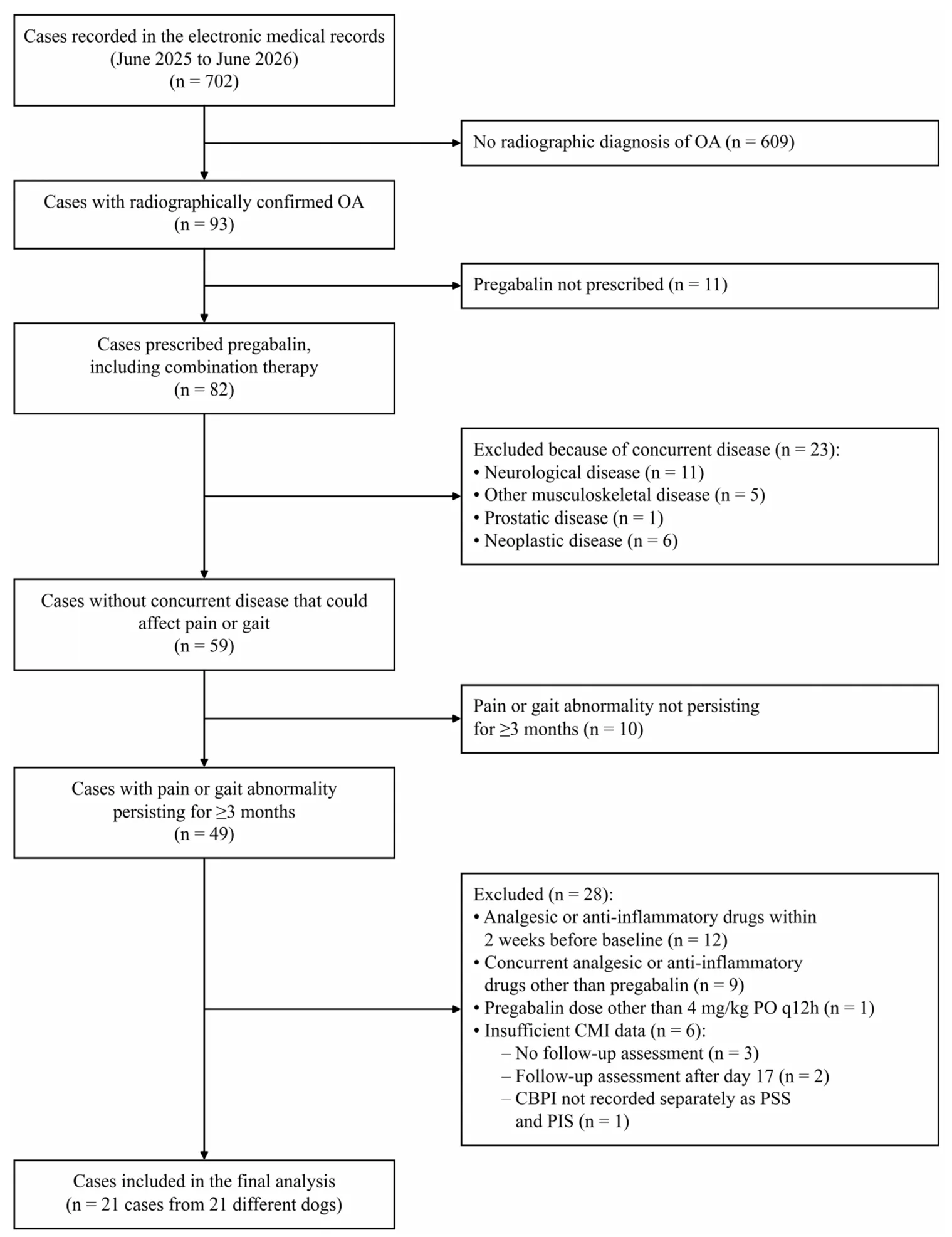
Flow diagram of case selection. Screening was performed at the case level, so a dog could contribute more than one case at the initial screening stages; the 21 cases included in the final analysis were from 21 different dogs. CBPI, Canine Brief Pain Inventory; CMI, clinical metrology instrument; OA, osteoarthritis; PIS, pain interference score; PSS, pain severity score.

**Figure 2:**
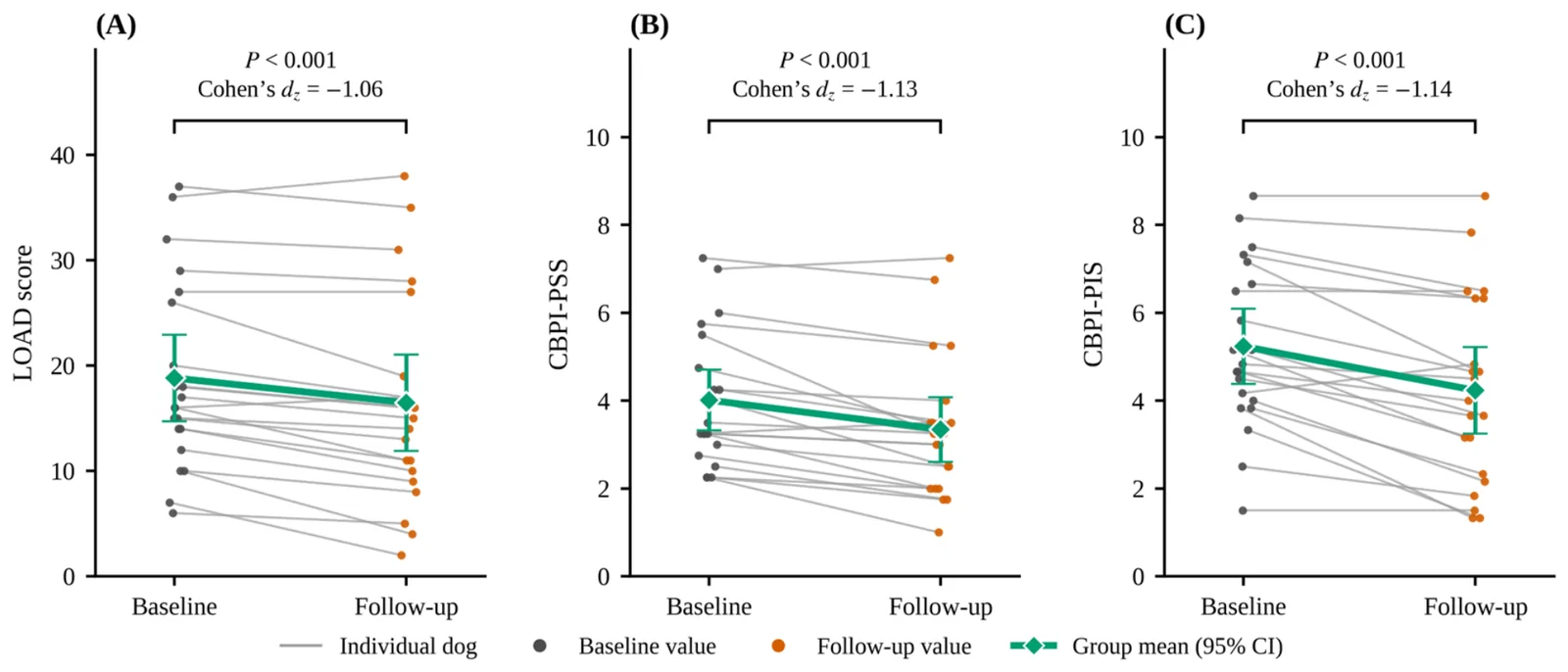
Handler-reported outcome measures in 21 working dogs with osteoarthritis at baseline and at follow-up 14 ± 3 days after initiation of pregabalin monotherapy (4 mg/kg PO q12h). (**A**) Liverpool Osteoarthritis in Dogs (LOAD) score; (**B**) Canine Brief Pain Inventory pain severity score (CBPI-PSS); (**C**) Canine Brief Pain Inventory pain interference score (CBPI-PIS). Gray lines connect the paired values of each dog, and dark gray and orange circles represent baseline and follow-up values, respectively. Green diamonds with error bars show the group mean and its 95% confidence interval. A small horizontal offset was applied to individual dogs to reduce overplotting; score values are unaltered. Brackets indicate the result of two-sided paired *t*-tests with the corresponding within-subject effect size (Cohen’s *d_z_*). For all three instruments, lower scores indicate less pain and better function.

**Figure 3:**
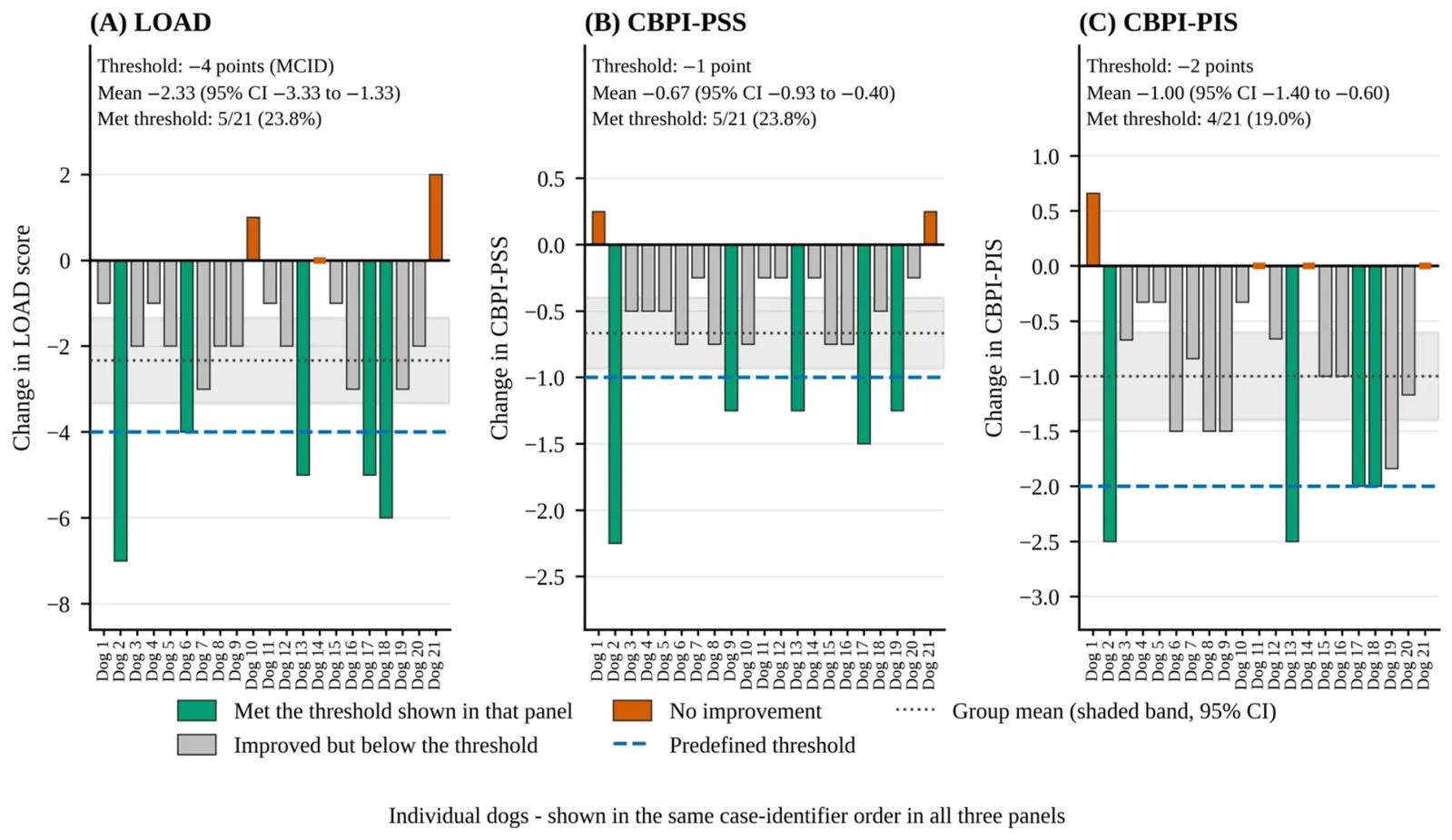
Individual changes in handler-reported outcome measures from baseline to follow-up in 21 working dogs with osteoarthritis receiving pregabalin monotherapy. (**A**) Liverpool Osteoarthritis in Dogs (LOAD) score; (**B**) Canine Brief Pain Inventory pain severity score (CBPI-PSS); (**C**) Canine Brief Pain Inventory pain interference score (CBPI-PIS). Each bar represents one dog and negative values indicate improvement. Dogs are arranged by case identifier in ascending order, and the same order is used in all three panels, so that a given position on the *x*-axis corresponds to the same dog throughout. Dashed blue lines mark the predefined thresholds: the published minimal clinically important difference of −4 points for LOAD, and the two components of the CBPI responder criterion (a reduction of ≥1 point in CBPI-PSS together with ≥2 points in CBPI-PIS). Dotted lines and shaded bands show the group mean change and its 95% confidence interval; in every panel the confidence interval lies entirely above the corresponding threshold. Green bars indicate dogs meeting the threshold shown in that panel, gray bars dogs that improved without reaching it, and orange bars dogs with no improvement. Dogs with no change are shown as flat markers at zero. Only Dog 2, Dog 13, and Dog 17 met the threshold in all three panels.

Figure 1, Figure 2 and Figure 3 were prepared with the assistance of generative AI tools. Figure 3 was generated from the authors’ own dataset with the assistance of Claude Opus 5 (Anthropic PBC, San Francisco, CA, USA; accessed on 19 August 2026), and Figure 1 and Figure 2 were generated from the authors’ own dataset with the assistance of Claude Opus 5.5 (Anthropic PBC; accessed on 28 September 2026). All figures were verified against the source data and the statistical output by the authors, who take full responsibility for the content of this publication.

## 3. Results

### 3.1. Study Population

The case selection process is shown in Figure 1. Of the 702 cases recorded during the study period, 93 had radiographically confirmed OA, and pregabalin, either alone or in combination with other drugs, had been prescribed in 82 of these cases. Of these, 23 cases were excluded because of concurrent disease (neurological disease, n = 11; other musculoskeletal disease, n = 5; prostatic disease, n = 1; neoplastic disease, n = 6), and 10 were excluded because the pain or gait abnormality had not persisted for at least three months. Of the remaining 49 cases, 28 were excluded because of analgesic or anti-inflammatory medication within two weeks before baseline (n = 12), concurrent analgesic or anti-inflammatory drugs other than pregabalin (n = 9), a pregabalin dose other than 4 mg/kg PO q12h (n = 1), or insufficient CMI data (n = 6). Although screening was performed at the case level, the 21 cases included in the final analysis were from 21 different dogs.

A total of 21 working dogs were included in the final analysis. By breed, there were 12 German Shepherd Dogs (57.1%), 6 Labrador Retrievers (28.6%), 2 English Springer Spaniels (9.5%), and 1 Belgian Malinois (4.8%). Biological sex was female in 5 dogs (23.8%) and male in 16 dogs (76.2%). Mean age was 8.29 ± 2.45 years, with a median of 7.0 years (IQR, 7.0–10.0) and a range of 4–14 years. Radiographic OA grade was grade 1 in 3 dogs (14.3%), grade 2 in 7 dogs (33.3%), grade 3 in 4 dogs (19.0%), and grade 4 in 7 dogs (33.3%). Monoarticular OA was present in 11 dogs (52.4%) and polyarticular OA in 10 dogs (47.6%).

### 3.2. Changes in LOAD and CBPI Scores Following Initiation of Pregabalin

The paired differences for all three CMIs showed no significant deviation from normality on the Shapiro–Wilk test (LOAD, *W* = 0.963, *p* = 0.574; CBPI-PSS, *W* = 0.923, *p* = 0.102; CBPI-PIS, *W* = 0.968, *p* = 0.683). Paired *t*-tests were therefore applied.

LOAD, CBPI-PSS, and CBPI-PIS scores were all significantly lower at follow-up than at baseline. LOAD decreased from 18.81 ± 9.01 to 16.48 ± 10.04 (mean change, −2.33; 95% CI, −3.33 to −1.33; *p* < 0.001; Cohen’s *d_z_* = −1.06), and CBPI-PSS decreased from 4.01 ± 1.52 to 3.35 ± 1.62 (mean change, −0.67; 95% CI, −0.93 to −0.40; *p* < 0.001; *d_z_* = −1.13). CBPI-PIS also decreased from 5.23 ± 1.88 to 4.23 ± 2.16 (mean change, −1.00; 95% CI, −1.40 to −0.60; *p* < 0.001; *d_z_* = −1.14). All three differences remained significant after Bonferroni correction and in the Wilcoxon signed-rank sensitivity analysis (all *p* < 0.001). Individual patterns of change are presented in Figure 2.

### 3.3. Clinical Responder Analysis, Adverse Effects, and Treatment at Follow-Up

According to the predefined responder criteria, 5 of 21 dogs (23.8%) were classified as responders for LOAD and 3 of 21 dogs (14.3%) for the CBPI (Figure 3). Three of 21 dogs (14.3%) met both the LOAD and the CBPI criteria. All three dogs meeting the CBPI criterion also met the LOAD criterion, and no dog met the CBPI criterion alone. All dogs had baseline scores that allowed the predefined reductions, except for one dog with a baseline CBPI-PIS of 1.50, which therefore could not meet the PIS component of the CBPI criterion; when this dog was excluded, 3 of 20 dogs (15.0%) met the CBPI criterion.

No clinical adverse effects, including sedation, ataxia, decreased appetite, vomiting, or diarrhea, were identified in the medical records of the 21 dogs included in the final analysis between the initiation of pregabalin and the follow-up assessment.

At the follow-up assessment, pregabalin had been administered for 14 days in 14 dogs, 15 days in 3 dogs, 12 days in 2 dogs, and 11 and 17 days in 1 dog each (median, 14 days; range, 11–17 days). Because OA-related pain was considered inadequately controlled with pregabalin alone, meloxicam was added to pregabalin at the follow-up visit in 18 of the 21 dogs (85.7%), with monitoring for adverse effects; meloxicam was not added in the three dogs that met both the LOAD and CBPI criteria. The decision to add meloxicam was based on the clinical judgment of the attending veterinarian as part of routine care, rather than on predefined or blinded criteria for treatment failure. Outcomes after the follow-up assessment were not analyzed.

### 3.4. Exploratory Subgroup Analyses

The results of the exploratory analyses are summarized in Table 1.

Differences in response between breeds were evaluated as an exploratory analysis. The study population comprised German Shepherd Dogs (n = 12), Labrador Retrievers (n = 6), English Springer Spaniels (n = 2), and a Belgian Malinois (n = 1); because the sample size for some breeds was very limited, comparisons across all breeds were considered to be of limited interpretability. Changes were therefore compared between German Shepherd Dogs and Labrador Retrievers, for which relatively larger numbers were available. In this comparison, the reduction in CBPI-PIS was significantly greater in Labrador Retrievers than in German Shepherd Dogs (median change, −1.92 vs. −0.67; *p* = 0.024), whereas changes in LOAD and CBPI-PSS did not differ significantly between the two breeds.

No significant differences in CMI changes were identified according to biological sex or pattern of joint involvement (monoarticular or polyarticular).

Age was not significantly associated with the change in any CMI (LOAD, ρ = −0.412, *p* = 0.064; CBPI-PSS, ρ = −0.266, *p* = 0.244; CBPI-PIS, ρ = −0.281, *p* = 0.217). Radiographic OA grade, likewise, was not significantly associated with changes in LOAD, CBPI-PSS, or CBPI-PIS.

## 4. Discussion

This study retrospectively evaluated changes in LOAD and CBPI scores from baseline to follow-up after the initiation of pregabalin monotherapy in working dogs with OA-associated chronic pain.

NSAIDs are currently the mainstay of pharmacological treatment for OA-related chronic pain in dogs, and multimodal analgesia—combining agents with different mechanisms of action—is used when required [6,13]. NSAIDs can cause gastrointestinal, renal, and hepatic adverse effects [28], and several circumstances common in working dogs may influence these risks in individual dogs. Exercise-associated gastritis and gastric ulceration have been reported as clinically important problems in working dogs performing high-intensity or sustained exercise [29], and the potential for additional gastrointestinal injury [30] may therefore warrant particular caution when NSAIDs are used in such dogs. In addition, because working dogs are active outdoors for prolonged periods and are exposed to the risk of trauma [1,29], dehydration, hypovolemia, and hemorrhage must be considered in this population. In dehydrated or hypovolemic states, the risk of renal adverse effects associated with NSAID use may increase [31], and some NSAIDs can alter platelet function in dogs [32]. These are documented risk factors rather than evidence that NSAID-associated adverse effects occur more frequently in working dogs than in companion dogs, but they suggest that analgesics with other mechanisms of action may be useful for individual dogs in which NSAIDs must be used with caution.

In dogs, clinical effects of pregabalin have been reported for neuropathic pain associated with Chiari-like malformation and syringomyelia and for pain management after surgery for intervertebral disc disease [12,33,34]. However, these studies did not evaluate OA-related chronic pain, and evidence for gabapentinoids in canine OA remains limited [15].

In the present study, LOAD, CBPI-PSS, and CBPI-PIS scores were significantly lower at follow-up than at baseline at the group level. However, because this was an uncontrolled before-and-after study, these changes cannot be attributed to pregabalin; they may also reflect natural fluctuation of clinical signs, regression to the mean, or expectation effects. Regression to the mean is possible because treatment is typically initiated when clinical signs are relatively severe, and expectation effects cannot be excluded because LOAD and the CBPI are based on behavior and function observed by owners or handlers [35,36,37] and the handlers were not blinded to treatment. The caregiver placebo effect in canine OA can be substantial: in a placebo-controlled study of dogs with OA-related lameness, it was observed in 56.9% of owner evaluations and in 39.7–44.8% of veterinarian lameness evaluations [38], and in a placebo-controlled trial that used a CBPI-based definition of treatment success similar to the responder criterion used in this study, 28.2% of placebo-treated dogs were classified as treatment successes at day 14 [39].

The most notable finding of this study is the discrepancy between statistical significance and clinical relevance. The mean change in LOAD (−2.33 points; 95% CI, −3.33 to −1.33) did not reach the proposed minimal clinically important difference of −4 points [26,27], and the mean changes in CBPI-PSS (−0.67 points) and CBPI-PIS (−1.00 points) were smaller than the reductions of 1 and 2 points, respectively, required by the CBPI responder criterion [25]; in each case, the entire 95% CI fell short of the corresponding threshold. Because the CBPI criterion is a responder definition for individual dogs rather than for group means [25], the individual-level results are more informative: only 5 of 21 dogs (23.8%) met the LOAD criterion, and 3 of 21 (14.3%) met the CBPI criterion. Meloxicam was added at the follow-up visit in 18 of the 21 dogs, but not in the three dogs that met both criteria. However, because the decision to add meloxicam was based on clinical judgment rather than on a blinded or predefined assessment of treatment failure, this association between responder status and subsequent meloxicam administration should be interpreted with caution. The results of this study should therefore be summarized as a statistically significant group-level reduction in scores, with clinically meaningful improvement in only a minority of individual dogs.

Several factors may have contributed to this discrepancy and should be addressed in future studies. First, the follow-up period was short, and the response was assessed at a single time point 14 ± 3 days after the initiation of pregabalin. In a placebo-controlled trial of grapiprant in dogs with OA, the CBPI treatment success rate in the grapiprant group continued to increase from day 14 to day 28 [39]; therefore, additional or delayed responses with longer treatment cannot be excluded. Second, pain phenotypes were not characterized, and disease severity was heterogeneous. Because pregabalin acts mainly on neuronal excitability and sensitization rather than on joint inflammation [10,11,13], dogs with predominantly inflammatory pain may respond less to pregabalin monotherapy; however, pain mechanisms were not investigated, and the sample was too small for stratified analyses. Future studies should therefore include longer treatment periods with repeated assessments and stratification by pain phenotype and disease stage, for example, using the Canine OsteoArthritis Staging Tool (COAST) [40].

In the exploratory subgroup analyses, Labrador Retrievers showed a greater reduction in CBPI-PIS than German Shepherd Dogs; however, because the sample size was limited and no correction was applied for multiple comparisons, this difference may have arisen by chance and should not be interpreted as a true breed-related difference in responsiveness to pregabalin. This observation should be regarded only as hypothesis-generating. Sex, age, number of affected joints, and radiographic OA grade were not significantly associated with changes in LOAD, CBPI-PSS, or CBPI-PIS, although the small sample size does not exclude such associations.

No adverse effects related to pregabalin were recorded in the medical records of the 21 dogs during the observation period. However, adverse effects were identified retrospectively from free-text records over a short period (11–17 days), so the absence of recorded adverse effects does not exclude their occurrence, particularly of mild effects such as sedation.

Other limitations related to the retrospective design should also be noted. The eligibility criteria excluded 28 of the 49 cases with chronic pain (Figure 1), which may have introduced selection bias. Because the data were collected during routine clinical care, pain arising from sources other than the radiographically affected joints cannot be completely excluded, and inter-observer agreement for radiographic grading could not be assessed. In addition, the modified Kellgren–Lawrence grading system has not been validated for joints other than the stifle, and COAST could not be applied retrospectively because the caregiver and veterinary assessments it requires were not systematically recorded; however, the radiographic grade was not an eligibility criterion and was used only for descriptive and exploratory analyses. Finally, adherence to pregabalin was not verified, and its brand and formulation, the body weight used for dose calculation, and whether handlers received standardized instructions for completing the CMIs were not recorded.

The principal contribution of this study is to provide preliminary observational data on changes in clinical metrology scores following the initiation of pregabalin monotherapy in working dogs, a population operating under distinctive conditions. These data do not establish clinical utility or support the substitution of pregabalin for established OA treatments. Multimodal management is a well-accepted approach to OA pain, and current guidelines include gabapentinoids as adjunctive options, particularly when sensitization or chronic neuropathic pain is suspected [13,15]. Given the limited proportion of responders in the present study, whether adjunctive pregabalin provides additional benefit to established OA treatments, or has a role when NSAIDs must be used with caution, should be investigated in controlled studies.

## 5. Conclusions

In working dogs with OA-related chronic pain, LOAD, CBPI-PSS, and CBPI-PIS scores were significantly lower at follow-up, 14 ± 3 days after the initiation of pregabalin monotherapy, than at baseline. However, the group-level mean changes did not reach the predefined clinical thresholds, and only a minority of individual dogs met the predefined responder criteria (23.8% for LOAD and 14.3% for the CBPI). Because this study was retrospective and uncontrolled, these changes cannot be attributed to pregabalin. The results should therefore be interpreted as preliminary observational data, and larger prospective controlled studies are required to determine the efficacy and safety of pregabalin, and potential predictors of individual response.

## Figures and Tables

**Table 1:** Exploratory subgroup and correlation analyses of changes in handler-reported outcome measures from baseline to follow-up in 21 working dogs with osteoarthritis receiving pregabalin monotherapy.

Factor and Group	n	LOAD Δ	CBPI-PSS Δ	CBPI-PIS Δ
***Breed* ^a^**				
German Shepherd Dog	12	−2.00 (−2.25, −1.00)	−0.50 (−0.75, −0.25)	−0.67 (−1.50, −0.33)
Labrador Retriever	6	−4.00 (−5.00, −3.00)	−1.00 (−1.25, −0.75)	−1.92 (−2.00, −1.21)
*p* value		0.086	0.085	**0.024**
***Sex***				
Female	5	−1.00 (−2.00, −1.00)	−0.50 (−0.75, −0.50)	−0.33 (−0.67, −0.33)
Male	16	−2.00 (−3.25, −1.75)	−0.63 (−0.88, −0.25)	−1.08 (−1.58, −0.58)
*p* value		0.294	>0.999	0.245
***Affected joints***				
Monoarticular OA	11	−2.00 (−5.00, −1.00)	−0.75 (−1.25, −0.38)	−1.50 (−2.00, −0.33)
Polyarticular OA	10	−2.00 (−3.00, −1.25)	−0.50 (−0.75, −0.25)	−0.92 (−1.13, −0.42)
*p* value		0.642	0.315	0.457
***Correlation, ρ (p value)* ^b^**				
Age	21	−0.412 (0.064)	−0.266 (0.244)	−0.281 (0.217)
Radiographic OA grade	21	0.395 (0.076)	−0.045 (0.846)	0.259 (0.257)

Δ, change from baseline (follow-up − baseline); negative values indicate improvement. Values are median (interquartile range) unless stated otherwise. Between-group comparisons were made with the Mann–Whitney U test. Bold indicates *p* < 0.05. ^a^ English Springer Spaniel (n = 2) and Belgian Malinois (n = 1) were not included in the breed comparison because of the small number of dogs. ^b^ Spearman rank correlation coefficient (ρ) for the association between the factor and the change in each outcome across all 21 dogs. CBPI-PIS, Canine Brief Pain Inventory pain interference score; CBPI-PSS, Canine Brief Pain Inventory pain severity score; LOAD, Liverpool Osteoarthritis in Dogs; OA, osteoarthritis.

## Data Availability

The data supporting the findings of this study are not publicly available because they were obtained from a military institution and are subject to institutional security policies. The data may be made available from the author upon reasonable request, subject to security review and approval by the relevant institution.

## Abbreviations

The following abbreviations are used in this manuscript:

CBPI	Canine Brief Pain Inventory
CI	Confidence Interval
CMI	Clinical Metrology Instrument
COAST	Canine OsteoArthritis Staging tool
IQR	Interquartile Range
LOAD	Liverpool Osteoarthritis in Dogs
NSAID	Non-Steroidal Anti-Inflammatory Drug
OA	Osteoarthritis
PIS	Pain Interference Score
PSS	Pain Severity Score
